# HIV-1 Tat Recruits HDM2 E3 Ligase To Target IRF-1 for Ubiquitination and Proteasomal Degradation

**DOI:** 10.1128/mBio.01528-16

**Published:** 2016-10-18

**Authors:** Anna Lisa Remoli, Giulia Marsili, Edvige Perrotti, Chiara Acchioni, Marco Sgarbanti, Alessandra Borsetti, John Hiscott, Angela Battistini

**Affiliations:** a Department of Infectious, Parasitic and Immune-Mediated Diseases, Istituto Superiore di Sanità, Rome, Italy; b National AIDS Center, Istituto Superiore di Sanità, Rome, Italy; c Istituto Pasteur-Fondazione Cenci Bolognetti, Rome, Italy

## Abstract

In addition to its ability to regulate HIV-1 promoter activation, the viral transactivator Tat also functions as a determinant of pathogenesis and disease progression by directly and indirectly modulating the host anti-HIV response, largely through the capacity of Tat to interact with and modulate the activities of multiple host proteins. We previously demonstrated that Tat modulated both viral and host transcriptional machinery by interacting with the cellular transcription factor interferon regulatory factor 1 (IRF-1). In the present study, we investigated the mechanistic basis and functional significance of Tat−IRF-1 interaction and demonstrate that Tat dramatically decreased IRF-1 protein stability. To accomplish this, Tat exploited the cellular HDM2 (human double minute 2 protein) ubiquitin ligase to accelerate IRF-1 proteasome-mediated degradation, resulting in a quenching of IRF-1 transcriptional activity during HIV-1 infection. These data identify IRF-1 as a new target of Tat-induced modulation of the cellular protein machinery and reveal a new strategy developed by HIV-1 to evade host immune responses.

## IMPORTANCE

Current therapies have dramatically reduced morbidity and mortality associated with HIV infection and have converted infection from a fatal pathology to a chronic disease that is manageable via antiretroviral therapy. Nevertheless, HIV-1 infection remains a challenge, and the identification of useful cellular targets for therapeutic intervention remains a major goal. The cellular transcription factor IRF-1 impacts various physiological functions, including the immune response to viral infection. In this study, we have identified a unique mechanism by which HIV-1 evades IRF-1-mediated host immune responses and show that the viral protein Tat accelerates IRF-1 proteasome-mediated degradation and inactivates IRF-1 function. Restoration of IRF-1 functionality may thus be regarded as a potential strategy to reinstate both a direct antiviral response and a more broadly acting immune regulatory circuit.

## INTRODUCTION

The complex pathogenesis of HIV-1 infection is determined in part by interactions between viral regulatory proteins and cellular factors that are responsible for both viral gene expression in different tissues and virus-induced physiological changes. The HIV-1 transactivator Tat is essential for efficient transcription of the integrated provirus and for efficient HIV-1 replication (1, 2). By specifically binding to the transactivation-responsive element region in the viral promoter, Tat enhances both transcription and transcriptional elongation (3). Independent of its ability to regulate HIV-1 transcription, Tat also contributes to viral persistence and dissemination by exerting a variety of other activities that directly or indirectly modulate the host antiviral immune response, including deregulation of cytokine expression (4), inhibition of dendritic cell maturation (5), suppression of antigen (Ag)-induced lymphocyte activation (6, 7), as well as activation of cell proliferation and increase of cell survival (8, 9). Tat protein is also released from acutely infected cells into the extracellular environment and taken up by neighboring noninfected cells, where similarly, it increases virus infectivity and modulates cellular functions (4, 10). Many of these functions depend on the ability of Tat to interact with host regulatory proteins and interfere with the expression of multiple cellular functions (11, 12).

Among the numerous Tat-interacting proteins, we previously demonstrated that Tat interacted with interferon regulatory factor 1 (IRF-1), the founding member of a family of nine transcriptional regulators that impacts various physiological functions, including the immune response to viral infection, oncogenesis, and development of an immune system (13–16). Although originally identified as a regulator of type I *IFN* gene expression, IRF-1 is not considered essential for *IFN* gene expression, except in cell-specific contexts (17–19). However, as an interferon (IFN)-regulated gene, IRF-1 is involved in IFN-induced antiviral immunity through the regulation of selected antiviral genes that cooperatively promote an effective antiviral program against a broad spectrum of viruses (20–22). By inducing a rapid IFN-independent expression of antiviral factors (18), IRF-1 thus provides a rapid antiviral defense upstream of the IRF3-activated IFN axis, that is particularly relevant for those pathogens, including HIV-1, that evade innate immunity by disrupting the induction and function of IFN. In addition to its antiviral activity, IRF-1 also impacts other aspects of immune regulation, including adaptive immunity and inflammation (23). IRF-1 is predominantly regulated at the transcriptional level (24, 25), but posttranslational modifications also play a significant, nonredundant role in the regulation of its activity (26–31). Like many other transcription factors, IRF-1 is a short-lived protein that is rapidly degraded via the ubiquitin-proteasome pathway (32, 33). The ubiquitination and degradation signals reside in the C-terminal portion of IRF-1 (32), and the degradation rate can be regulated in response to cellular conditions and specific stress (34, 35).

In the context of HIV-1 infection, IRF-1 can act both as an inducer of viral gene expression and as an antiviral factor, depending on the physical interactions between Tat and IRF-1 in HIV-1-infected cells. In particular, during the early phase of infection, IRF-1 is induced by HIV-1 and, in combination with NF-κB, activates proviral transcription irrespective of the presence of Tat. Later, when discrete amounts of Tat are produced and IRF-1 becomes dispensable for long terminal repeat (LTR) activity, interaction with Tat sequesters IRF-1, resulting in the quenching of its transcriptional activity on target genes (36–39).

In the present study, we have examined the mechanistic basis of IRF-1 expression modulation by viral Tat and now demonstrate that Tat targets IRF-1 for ubiquitin-mediated, K48-dependent proteasome degradation. We also identify human double minute 2 protein (HDM2) as the IRF-1-specific ligase utilized by Tat to decrease IRF-1 stability. HDM2 (also known as mouse double minute 2 [Mdm2] in mice) is an E3 ubiquitin ligase that ubiquitinates the tumor suppressor p53 and is required for proteasome-dependent degradation and nuclear export of p53 (40, 41). HDM2 also targets other viral and cellular substrates (40), including members of the IRF family (42, 43), although the physiological significance of HDM2-IRF interactions has not been fully addressed. Our observations identify an additional mechanism by which HIV-1 may suppress the antiviral immune response and contribute to immune dysfunctions that favor viral replication and disease progression*.*

## RESULTS

### Tat affects IRF-1 protein stability.

**FIG 1:**
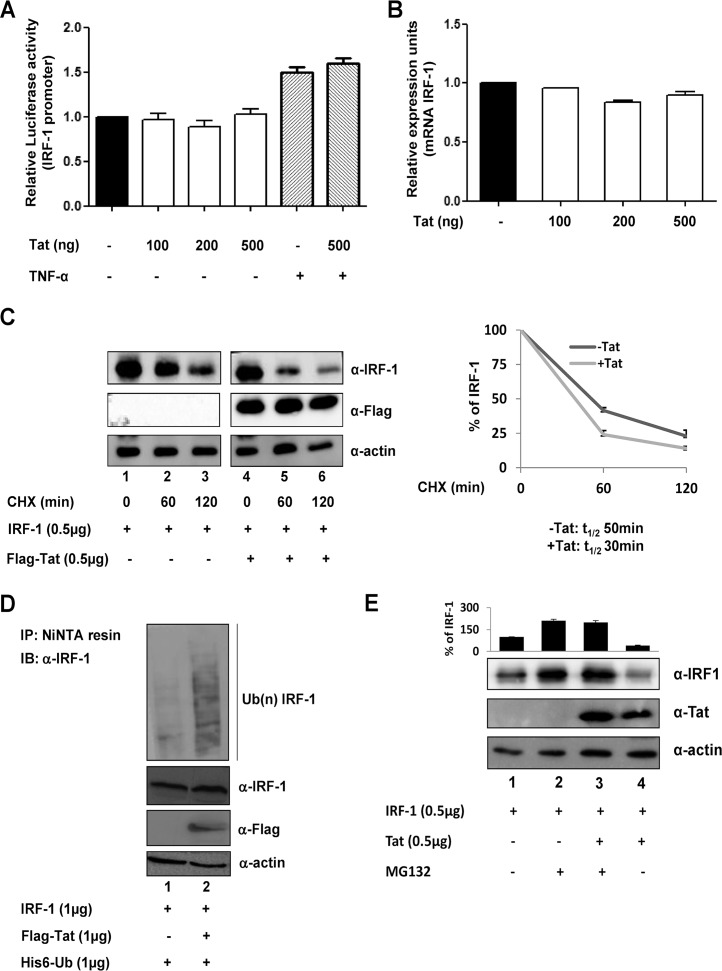
Tat affects IRF-1 stability. (A) HEK293 cells were transfected with a 3,500-bp fragment of the IRF-1 promoter linked to the luciferase reporter gene alone or in combination with increasing amounts of Tat-expressing vector. After 24 h, cells were treated for 4 h with 10 ng/ml of TNF-α (+), where indicated, and then processed for luciferase activity. Data shown are the means plus standard errors of the means (SEM) (error bars) from three separate experiments calculated after normalization with the *Renilla* activity. The values for untreated cells were set at 1. (B) HEK293 cells were transfected with increasing amounts of Tat-expressing vector. At 24 h after transfection, cells were harvested, and IRF-1 RNA levels were assessed using real-time RT-PCR. The levels were normalized to GAPDH mRNA abundance. The means plus SEM of three independent experiments are shown as relative expression units. The values for untreated cells were set at 1. (C) HEK293 cells were transfected with IRF-1 expression vector in the presence (+) or absence (−) of Flag-Tat expression vector. At 24 h posttransfection, the cells were treated with CHX for the indicated time. IRF-1 and Tat proteins were detected with anti-IRF-1 (α-IRF-1) and anti-Flag (α-Flag) antibodies, respectively. Data plotted in the graph represent the means ± SEM from three different assays of IRF-1 protein bands quantified from Western blots and normalized to actin protein levels as the loading control and presented as percentage values relative to those without CHX treatment set at 100%. (D) HEK293 cells were cotransfected with expression vectors for His6-Ub and IRF-1 in the presence or absence of Flag-Tat-expressing vector. His-Ub-conjugated proteins were captured by nickel-agarose beads, eluted, and analyzed by Western blotting with anti-IRF-1 antibody. Western blotting of cell lysates shows the expression of ectopically expressed proteins. (E) HEK293 cells were cotransfected with expression vectors for IRF-1 and Flag-Tat and then treated with MG132 for 2 h where indicated. IRF-1 and Tat expression was detected by Western blotting. Data plotted in the graph represent the means plus SEM from three different assays of IRF-1 protein bands quantified from Western blots and normalized to actin protein levels as the loading control. Results are presented as percentage values relative to basal IRF-1 expression set at 100. Blots are representative of at least three independent experiments with similar results.

In previous studies, we reported that HIV-1 Tat physically interacted *in vitro* and *in vivo* with IRF-1 (37–39), and we also observed that coexpression of Tat and IRF-1 cause a reproducible decrease in IRF-1 accumulation. Because IRF-1 expression is primarily regulated at the transcriptional level, the effect of Tat on IRF-1 transcription was initially evaluated. Analysis of the transcriptional activity of a 3,500-bp fragment of the IRF-1 promoter linked to the luciferase reporter gene indicated that the basal and tumor necrosis factor alpha (TNF-α)-stimulated IRF-1 promoter activity was not affected by increasing amounts of Tat expression compared with cells expressing an empty vector (Fig. 1A). Similarly, no variation in IRF-1 mRNA levels was observed in the presence of increasing Tat (Fig. 1B). Therefore, to determine whether Tat could modulate IRF-1 protein stability, IRF-1 was coexpressed together with Flag-tagged Tat in the presence of the protein synthesis inhibitor cycloheximide (CHX). In the presence of Tat, a significant acceleration of IRF-1 decay was detected at 24 h posttransfection (Fig. 1C, lanes 5 and 6 versus lanes 2 and 3). Densitometric quantification of protein levels indicated that in the absence of Tat expression, the half-life of IRF-1 was 50 min, whereas in the presence of Tat, IRF-1 protein half-life was reduced to ~30 min. Since it is known that IRF-1 is degraded through the ubiquitin/proteasome pathway, we wondered whether Tat could stimulate IRF-1 polyubiquitination and proteasome degradation. IRF-1 and six-histidine-tagged ubiquitin (His6-Ub) were coexpressed in the presence or absence of Flag-tagged Tat. IRF-1 ubiquitination was then determined by capturing His6-Ub in cell extracts with nickel beads (nickel-nitrilotriacetic acid [Ni-NTA]), followed by Western blot analysis of the purified ubiquitin conjugates with IRF-1-specific antibodies. Monoubiquitinated IRF-1 was detected in the His-Ub-expressing cells, while IRF-1 polyubiquitination was easily detected in Tat-containing extracts (Fig. 1D). To assess the proteasome-mediated IRF-1 degradation, IRF-1 expression was analyzed in cells expressing IRF-1 alone or in combination with Flag-tagged Tat in the presence or absence of the proteasome inhibitor MG132. MG132 clearly blocked the ability of Tat to accelerate IRF-1 turnover (Fig. 1E, lane 3 versus lane 4), suggesting that Tat may indeed increase the proteasome-mediated degradation of IRF-1.

### HDM2 E3 ligase mediates Tat-induced IRF-1 turnover.

**FIG 2:**
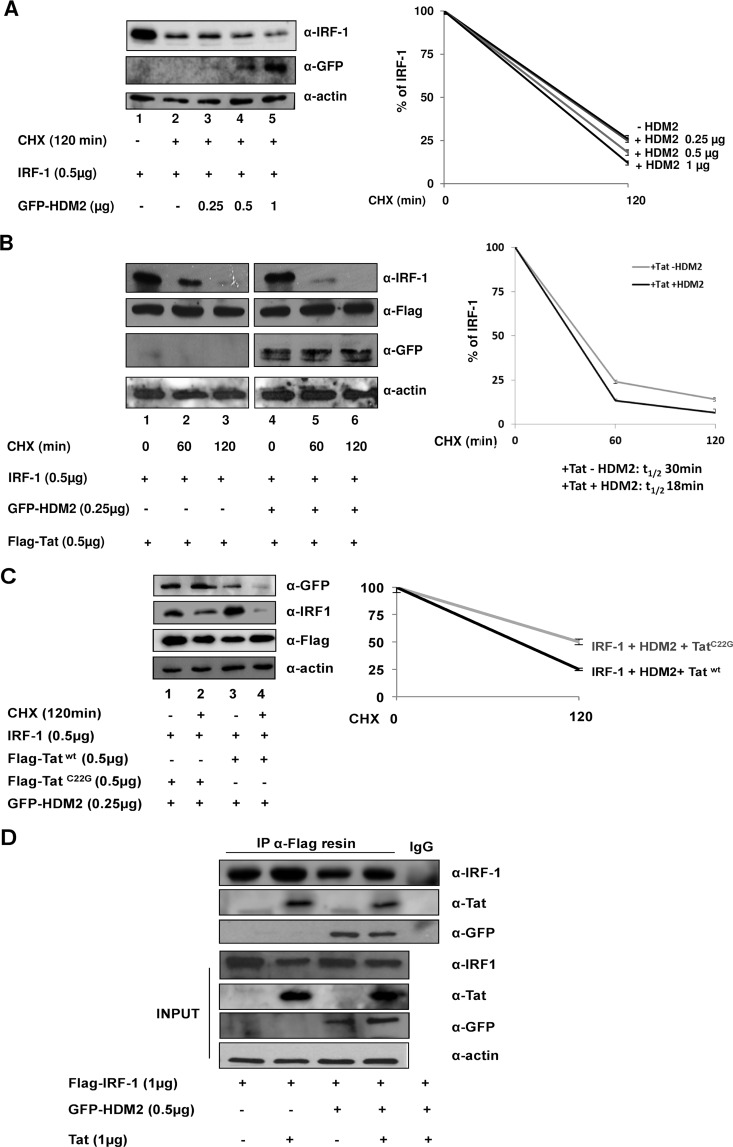
HDM2 E3 ligase mediates Tat-induced IRF-1 turnover. (A) HEK293 cells were transfected with expression vectors encoding IRF-1 alone or in combination with increasing amounts of GFP-tagged HDM2 (GFP-HDM2), as indicated. The cells were treated with CHX for the indicated time, and cell lysates were then subjected to immunoblotting. Data plotted in the graph represent the means ± SEM from three different assays of IRF-1 protein bands quantified from Western blots and normalized to actin protein levels as the loading control and presented as percentage values relative to those without CHX treatment set at 100. (B) HEK293 cells were transfected with expression vectors for IRF-1, Flag-Tat, and HDM2-GFP expression vector as indicated. The cells were then treated with CHX, and IRF-1 expression was assessed by immunoblotting. Data plotted in the graph are calculated and presented as in panel A. (C) HEK293 cells were transfected with expression vectors encoding IRF-1, GFP-HDM2, and wild-type Flag-Tat (Flag-Tat^wt^) or Flag-Tat^C22G^. The cells were then treated with CHX, and IRF-1 expression was assessed as described above for panel A. (D) HEK293 cells were transfected with expression vectors for Flag-IRF-1 and Tat and/or GFP-HDM2, as indicated. Whole-cell extracts were incubated with anti-Flag (α-Flag)-conjugated resin or control IgG, and immunoprecipitated (IP) complexes were separated by SDS-PAGE and subsequently probed with anti-Tat, anti-GFP, or anti-IRF-1 antibodies, respectively. The levels of ectopically expressed proteins are shown in the INPUT blots.

In searching for the E3 ubiquitin ligase that mediates Tat-induced polyubiquitination/degradation of IRF-1, HDM2 was selected for further investigation, given previous studies that established a link between HDM2 and both Tat (44) and IRF-1 (42). HDM2 was reported to interact with Tat as an E3 ligase that increases Tat-mediated transactivation of the LTR upon K63 ubiquitination in Tat-expressing cells (44). Similarly, HDM2 was shown to bind and ubiquitinate IRF-1 (42). We therefore initially examined whether HDM2 affected IRF-1 stability. In the presence of CHX and increasing amounts of green fluorescent protein (GFP)-tagged HDM2, HDM2 *per se* decreased IRF-1 levels in a dose-dependent manner (Fig. 2A, lanes 4 and 5 and graph). Then, to assess whether the Tat-mediated decrease in IRF-1 stability involved HDM2, IRF-1 was coexpressed together with Flag-tagged Tat using small amounts of HDM2 that did not *per se* affect IRF-1 stability. In the presence of Tat, IRF-1 degradation was dramatically accelerated by HDM2 coexpression compared to cells not expressing HDM2 (Fig. 2B, lanes 5 and 6 versus lanes 2 and 3). Densitometric quantification of protein levels indicated that in the absence of HDM2 expression, the half-life of IRF-1 was ~30 min, whereas in the presence of HDM2, the half-life of IRF-1 protein was reduced to ~18 min.

The specificity of the effect of Tat on IRF-1 stability was then evaluated in expression studies using a mutation in cysteine 22 (Tat^C22G^) of Tat that reduces both interaction with HDM2 and Tat ubiquitination (44). Compared with the effect of wild-type Tat, the Tat^C22G^ mutant was unable to accelerate HDM2-mediated IRF-1 degradation (Fig. 2C, lane 2 versus lane 4).

In support of the interrelationship between Tat, IRF-1, and HDM2, coimmunoprecipitation experiments with Flag-tagged IRF-1, GFP-tagged HDM2, and Tat indicated a physical association of the three proteins (Fig. 2D). Collectively, these results indicate that Tat accelerates IRF-1 turnover upon recruitment of the HDM2 E3 ligase.

### **Tat increases HDM2-induced IRF-1 K48 polyubiquitinatio**n.

**FIG 3:**
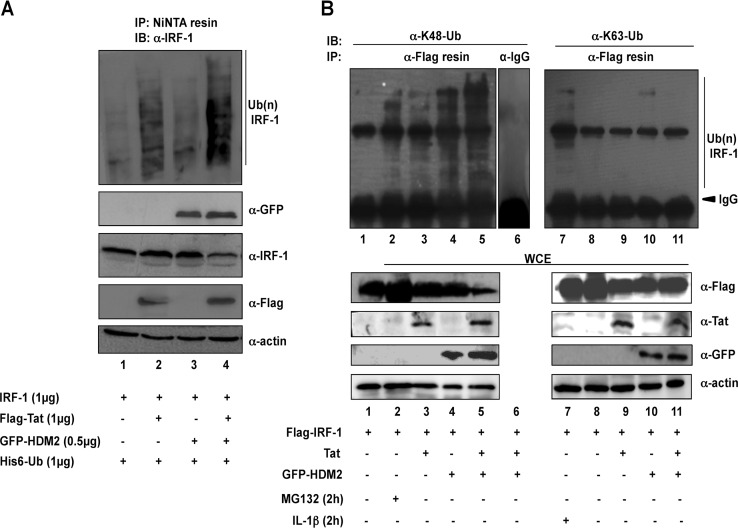
Tat increases HDM2-mediated K48 polyubiquitination of IRF-1. (A) IRF-1 ubiquitination in the presence of Flag-Tat, GFP-HDM2, or both was monitored as described in the legend to Fig. 1D. Western blots show the expression, in whole-cell extracts (WCE), of ectopically expressed proteins. (B) HEK293 cells were cotransfected with Flag-tagged IRF1, GFP-HDM2, and Tat alone or in combination, as indicated. Two-hour treatment with MG132 (lane 2) or IL-1β (lane 7) was used for positive internal controls. Immunoprecipitation (IP) with Flag-conjugated resin was performed, and the IRF1 ubiquitination forms were detected by Western blotting or immunoblotting (IB) with anti-K48-linked ubiquitin (α-K48-Ub) or anti-K63-linked ubiquitin (α-K63-Ub) or control IgG. Western blots show the expression, in whole-cell extracts, of ectopically expressed proteins.

The nickel capture assay was next used to evaluate the effect of Tat on HDM2-dependent IRF-1 ubiquitination; expression of either Tat or HDM2 stimulated the accumulation of the ubiquitinated IRF-1 (Fig. 3A, lanes 2 and 3), and the coexpression of Tat together with HDM2 greatly increased the accumulation of the polyubiquitinated forms of IRF-1 (Fig. 3A, lane 4). The low level of IRF-1 ubiquitination present in the control extract (Fig. 3A, lane 1) is likely mediated by endogenous E3-ligase activity. Since ubiquitination is not limited to proteasomal degradation, we next determined whether degradation of IRF1 by Tat and HDM2 involved the formation of K48-linked polyubiquitination chains that act as a *bona fide* signal for targeting substrates to proteasomal degradation. Using Flag-tagged IRF1 with GFP-tagged HDM2 in the presence or absence of Tat, immunoprecipitation was performed using an anti-Flag antibody-conjugated resin, and IRF-1-linked polyubiquitination chains were detected using antibodies specific for K48- or K63-linked ubiquitin, respectively. Both Tat and HDM2 individually induced K48-linked IRF-1 ubiquitination (Fig. 3B, lanes 3 and 4), while Tat and HDM2 together further increased K48 ubiquination of IRF-1 (Fig. 3B, lane 5). In contrast, no K63-linked ubiquitin chains were observed under the same conditions (Fig. 3B, lanes 8 to 11), whereas K63-linked IRF-1 polyubiquitination was detected in cells stimulated with IL-1β (Fig. 3B, lane 7). 

### Tat-mediated IRF-1 degradation requires the IRF-1 C-terminal domain. 

**FIG 4:**
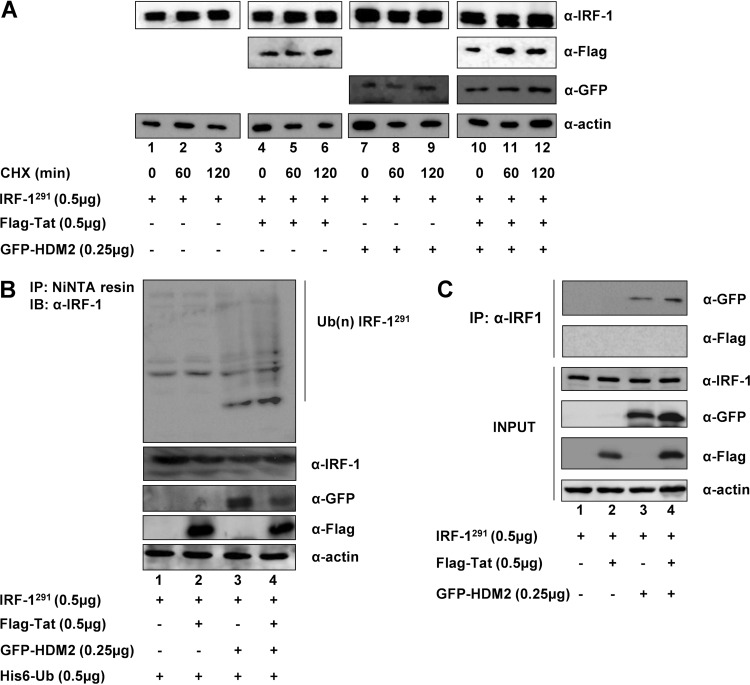
Tat-mediated IRF-1 degradation requires the IRF-1 C-terminal domain. (A) HEK293 cells were cotransfected with expression vectors for an IRF-1 mutant with the 34-aa COOH terminus deleted (IRF1^291^), Flag-Tat, and GFP-HDM2, alone or in combination. One day after transfection, the cells were treated with CHX for the indicated time points, and expression of IRF1^291^, Tat, and HDM2 was detected by Western blotting using specific antibodies, as indicated. (B) IRF1^291^ ubiquitination in the presence of Flag-Tat, GFP-HDM2, or both, was monitored as described in the legend to Fig. 3A. Western blots show the expression of ectopically expressed proteins in whole-cell extracts. (C) HEK293 cells were cotransfected with the indicated expression vectors, immunoprecipitated with anti-IRF-1 antibodies, and Tat and HDM2 were detected by Western blotting using the indicated antibodies. INPUT shows the level of ectopically expressed proteins.

The motifs required for polyubiquitination and proteasomal degradation of IRF-1 were previously identified in the C-terminal domain, spanning nucleotides 255 to 325 (32). Consistent with these observations, a deleted version of IRF-1 lacking the C-terminal 34-amino-acid (aa)  sequence (IRF-1^291^), showed no change in stability, either alone or in the presence of Tat (Fig. 4A, lanes 1 to 6). Furthermore, when IRF-1^291^ was coexpressed with GFP-tagged HDM2 expression vector alone (Fig. 4A, lanes 7 to 9) or together with Flag-tagged Tat (Fig. 4A, lanes 10 to 12), IRF-1^291^ expression was unchanged. Consistent with the observation that IRF-1 residues involved in ubiquitination are distinct from residues required for degradation (32), the nickel capture assay, used to evaluate ubiquitination of mutated IRF-1, indicated that HDM2 was still able to ubiquitinate IRF-1^291^ (Fig. 4B, lane 3), whereas Tat did not induce IRF-1^291^ ubiquitination alone or in the presence of HDM2 (Fig. 4B, lanes 2 and 4). This result is consistent with the observation that the C-terminal 34 aa of IRF-1 is required for the interaction with Tat (37). Coimmunoprecipitation experiments with Flag-tagged Tat, GFP-tagged HDM2, and IRF-1^291^ confirmed that the C-terminal 34 aa of IRF-1 is required for the interaction with Tat but dispensable for the binding to HDM2 (Fig. 4C, lanes 2 and 3). Collectively, these results indicate that the 34-aa C-terminal portion of IRF-1 was required for the increased IRF-1 turnover upon interaction with Tat.

### Inhibition of IRF-1 transcription by HDM2 is accelerated in the presence of Tat.

**FIG 5:**
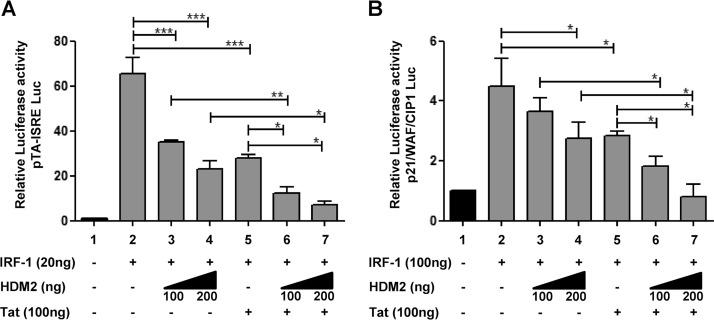
Inhibition of IRF-1-dependent transcription by HDM2 is accelerated in the presence of Tat. Transcription of the IRF-1-responding constructs pTA-ISRE-Luc (Luc stands for luciferase) (A) and the human *p21* gene promoter linked to the luciferase reporter gene (B) was measured by dual-luciferase assay in whole-cell extracts from HEK293 cells 24 h after transfection with expression vectors for IRF-1, Tat, and HDM2 as indicated. Means plus standard deviations (SD) from three separate experiments were calculated after normalization with the *Renilla* activity are shown. Values that are significantly different are indicated by bars and asterisks as follows: *, *P* < 0.05; **, *P* < 0.01; ***, *P* < 0.001.

To address the functional consequences of IRF-1 turnover, we next examined how expression of Tat and HDM2 affected IRF-1-dependent gene expression. The transcriptional activity of IRF-1-responsive luciferase reporter constructs pISRE-TA (bearing five copies of the consensus IRF-E motif) and the IRF-1-responsive *p21*^WAF/CIP1^ gene promoter was evaluated in HEK293 cells transiently cotransfected with IRF-1 and increasing amounts of HDM2 in the presence or absence of Tat (Fig. 5A and B). Expression of the IRF-1-responsive promoters was reduced ~50 percent by Tat expression, and increasing amounts of HDM2 also decreased IRF-1 promoter expression by 20% to 70%; in the presence of both Tat and HDM2, IRF-1 driven promoter activity was essentially abolished and returned to basal levels. In contrast, IRF-1^291^ was not affected by Tat alone or in combination with HDM2 (data not shown).

### IRF-1 degradation is increased in Jurkat cells inducibly expressing Tat protein.

**FIG 6:**
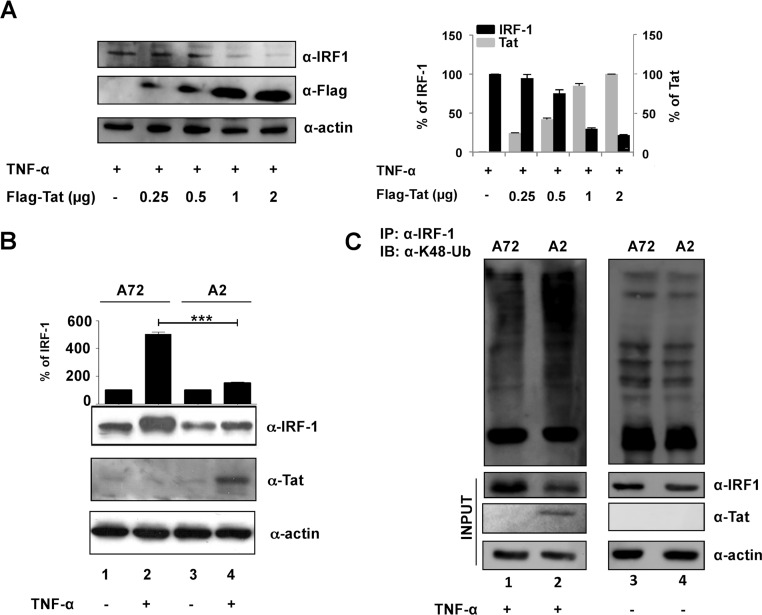
IRF-1 degradation is increased in cells expressing Tat protein. (A) HEK293 cells were transfected with the indicated doses of expression vector encoding Flag-Tat, and one day after transfection, the cells were treated with 10 ng/ml of TNF-α for 4 h. Cell lysates were then subjected to immunoblotting using anti-IRF-1 and anti-Flag antibodies. Data plotted in the graph represent the means plus SEM from three different assays of IRF-1 and Tat protein bands quantified from Western blots and normalized to actin protein levels as the loading control. Results are presented as percentage values relative to IRF-1 expression in control TNF-treated cells and ectopically expressed Tat (2 µg) set at 100. (B) Jurkat A2 cells were induced by TNF-α for 48 h to express Tat, and the levels of IRF-1 and Tat proteins were determined by Western blotting analysis using specific anti-IRF-1 and anti-Tat antibodies, respectively. Means plus SD from three separate experiments calculated after normalization with actin and with the control set at 100% are shown (***, *P* < 0.001). (C) Endogenous IRF-1 protein was immunoprecipitated using anti-IRF-1 antibody from A72 and A2 Jurkat cell lines treated with TNF-α (+) or not treated with TNF-α (−). IRF-1 ubiquitination was detected upon blotting with anti-K48 ubiquitin antibody (α-K48). The levels of ectopically expressed proteins are shown in the INPUT blots.

To evaluate whether Tat also decreased accumulation of endogenous IRF-1, we transfected a Tat-expressing construct in cells where expression of endogenous IRF-1 was stimulated by TNF-α Increasing amounts of Tat were indeed able to affect IRF-1 expression in a dose-response manner (Fig. 6A and graph). Moreover, the Tat-mediated IRF-1 proteolysis was also evaluated in a more physiologically relevant cell model, i.e., in a Jurkat T cell clone (termed A2) that inducibly expresses Tat following TNF-α treatment (45). The content of IRF-1 in control A72 and in Tat-expressing A2 cells was thus evaluated, and as previously reported, TNF-α treatment stimulated IRF-1 expression in A72 control cells (Fig. 6B, lane 2 and graph). Conversely, in Tat-expressing A2 cells, IRF-1 did not accumulate (Fig. 6B, lane 4 and graph). Importantly, in A2 cells, Tat expression following TNF-α treatment resulted in K48-linked ubiquitination of IRF-1 (Fig. 6C, lane 2) compared to A72 cells that do not express Tat (Fig. 6C, lane 1). As expected, similar basal levels of IRF-1 ubiquitination were observed in the two cell lines in the absence of TNF-α treatment (Fig. 6C, lanes 3 and 4).

### IRF-1 is downregulated and K48 polyubiquitinated in HIV-1-infected Jurkat T cells and during HIV-1 *de novo* infection of human primary CD4^+^ T cells.

**FIG 7:**
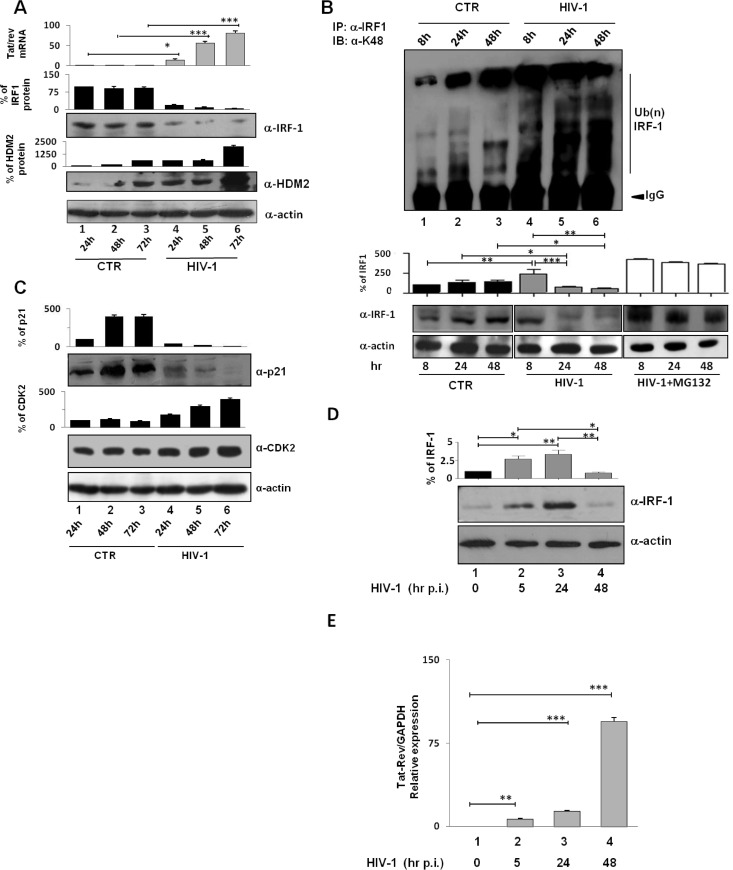
IRF-1 is downregulated and K48 polyubiquitinated in HIV-1-infected Jurkat T cells and during HIV-1 *de novo* infection of human primary CD4^+^ T cells when Tat is maximally expressed. (A, top) Tat/Rev RNA levels were measured by real-time RT-PCR as described in Materials and Methods. Means plus SD from three separate experiments calculated after normalization with GAPDH are shown (*, *P* < 0.05; ***, *P* < 0.001). (Bottom) WCE were prepared at the indicated time points from infected and uninfected Jurkat cells and then probed with anti-IRF-1, anti-HDM2, and anti-actin antibodies, respectively. Representative Western blots are shown. Data plotted in the graphs represent the means ± SEM from three different assays of IRF-1 and HDM2 protein bands quantified from Western blots and normalized to actin protein levels as the loading control (CTR). Results are presented as percentage values relative to basal IRF-1 and HDM2 expression set at 100. (B) WCE were prepared at the indicated time points from control and HIV-1-infected cells and immunoprecipitated with anti-IRF-1 antibody. IRF-1 ubiquitinated forms were detected using anti-K48 ubiquitin antibody (α-K48). The Western blot is representative of at least three independent experiments with similar results. (Bottom) The levels of IRF-1 in control cells, HIV-1-infected cells, and HIV-1-infected cells in the presence of MG132 are shown. Data plotted in the graphs are the means plus SD of IRF-1-specific bands quantified from Western blots normalized to actin from three independent experiments (*, *P* < 0.05; **, *P* < 0.01; ***, *P* < 0.001). (C) WCE as in panel A were probed with anti-p21 and anti-CDK2 antibodies, respectively. (Top) Quantification of p21 and CDK2 calculated as in panel A. (Bottom) Representative Western blots. (D) Purified human primary CD4^+^ T cells were infected with HIV-1 as described in Materials and Methods, and WCE were subjected to Western blot analysis with specific anti-IRF-1 antibody. Data plotted in the graph represent the means plus SD from three independent experiments (*, *P* < 0.05; **, *P* < 0.01). The time (in hours postinfection [hr p.i.]) is shown below the blot. (E) Total RNA was extracted at the indicated time points from cells as described above for panel D and analyzed by real-time RT-PCR for the doubly spliced (Tat/Rev) transcript as described in Materials and Methods (**, *P* < 0.01; ***, *P* < 0.001).

To assess the biological relevance of the above findings, the turnover of IRF-1 was also evaluated in the context of HIV-1 infection. In HIV-infected Jurkat T cells beginning 24 h postinfection, IRF-1 expression was substantially decreased (Fig. 7A, lanes 4 to 6 versus lanes 1 to 3), while HDM2 expression increased during the course of infection (Fig. 7A). The turnover of IRF-1 expression mirrored the increase in Tat/Rev transcripts (Fig. 7A, top panel). Detection of IRF-1-linked polyubiquitination chains indicated that K48-linked ubiquitination of IRF-1 was substantially increased in HIV-1-infected cells beginning 8 h postinfection compared with control cells (Fig. 7B, lanes 4 to 6 versus lanes 1 to 3). Furthermore, with increased K48 ubiquitination, IRF-1 protein levels dramatically decreased, whereas this reduction was absent when infection was performed in the presence of MG132 (Fig. 7B, bottom blots and graphs). Finally, from 24 h postinfection onward, the transcriptional activity of IRF-1 in HIV-1-infected cells was also impaired as measured by the expression of the IRF-1-regulated genes p21^WAF/CIP1^ and CDK2. The expression of p21^WAF/CIP1^ was dramatically downregulated with time after infection, whereas expression of CDK2, a gene negatively regulated by IRF-1, was increased more than 2-fold (Fig. 7C).

IRF-1 expression was then evaluated after *de novo* HIV-1 infection of primary human CD4^+^ T cells; an early 2- to 3-fold stimulation of IRF-1 expression, was followed by an inhibition of IRF-1 expression to levels present in uninfected cells (Fig. 7D). Interestingly, the lowest levels of IRF-1 correlated with the highest levels of the double-spliced (Tat/Rev) viral transcripts (Fig. 7E) and K48-linked ubiquitination of IRF-1 (data not shown). Collectively, these results reveal that IRF-1 is dramatically downmodulated by increasing amounts of Tat, suggesting an active role of Tat in modulating IRF-1 expression and IRF-1-induced signature in T cells during HIV-1 infection.

## DISCUSSION

**FIG 8:**
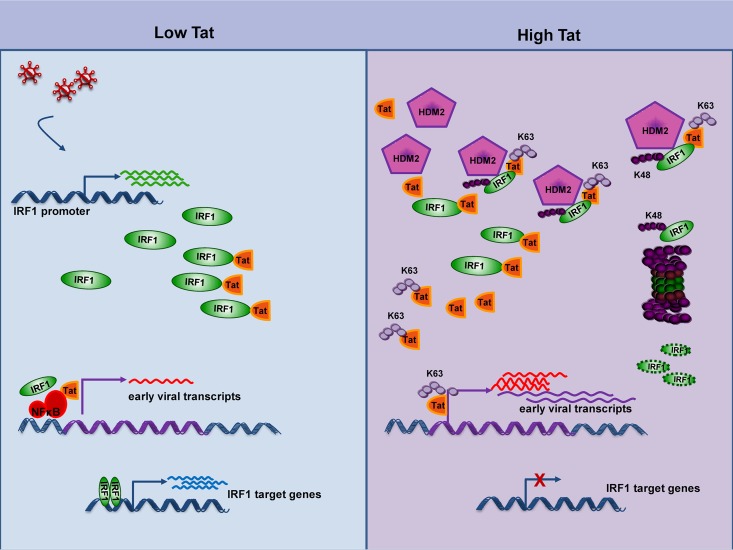
Schematic representation of the dual effect of Tat on IRF-1 activity in the course of HIV-1 infection. In early phases of HIV-1 replication, IRF-1 is transcriptionally stimulated by viral infection, and it is recruited by small amounts of Tat on the viral promoter to drive, with NF-κB, initial transcription of the integrated provirus. Later, when discrete amounts of Tat are produced and IRF1 activity on LTR is dispensable for the virus to replicate, Tat nullifies the function of IRF-1, accelerating its proteasome-mediated degradation upon recruitment of the HDM2 E3 ligase, thus quenching its activity on target gene promoters.

In previous studies, we demonstrated that the host transcription factor IRF-1 is utilized early after *de novo* HIV-1 infection to initiate proviral transcription in the absence of expression of the viral Tat protein. At later times after infection, when discrete amounts of Tat are produced and available to amplify proviral transcription, IRF-1 becomes dispensable for proviral gene expression and is specifically targeted by Tat, resulting in IRF-1 inactivation (37–39). We have now extended these studies to demonstrate that HIV-1 Tat targets IRF-1 for degradation by recruiting the HDM2 E3 ligase to IRF-1, catalyzing K48-linked polyubiquitination that leads to proteasome-mediated degradation. Tat thus contributes to the inhibition of IFN antiviral signaling by inactivation of IRF-1 target gene expression (Fig. 8).

IRF-1 activity is regulated both at the transcriptional level (24, 25) and via posttranslational modifications that include regulatory phosphorylation, sumoylation, acetylation, ubiquitination, and proteasomal degradation (26–31). In more general terms, protein ubiquitination is an effective and rapid mechanism to modulate antiviral signaling and trigger a host response against RNA or DNA viruses (46, 47). Both K48-linked polyubiquitination, leading to proteasomal degradation, and K63-linked polyubiquitination, modulating nonproteolytic processes, contribute to this regulation (48–51). A number of adaptor signaling proteins in the pathogen-sensing pathways are activated by K63-linked ubiquitination (52, 53), including IRF-1 in response to interleukin 1β (IL-1β) (54), while ubiquitin-mediated degradation of IRF-3, IRF-7, and IRF-5 is an effective mechanism to dampen host antiviral response (55). As such, these modifications are exploited by a number of viruses, including HIV-1, to block the innate immune response to infection (56–59).

Although IRF-1 has been characterized as a substrate of the ubiquitination machinery, an E3 ligase capable of mediating IRF-1 ubiquitination during virus infection had not been identified. In addition to the identification of HDM2 as the E3 ligase recruited to IRF-1 by Tat, we also demonstrate that HDM2 mediated K48-linked ubiquitination of IRF-1 and represented a signal for IRF-1 proteolysis. IRF-1 ubiquitination by HDM2 was reported previously (42), although IRF-1 degradation was not observed, and it was concluded that HDM2 was involved in regulation of IRF-1 activity, rather than rate of degradation. It should be noted that IRF-1 expression and stability may be highly divergent depending on the model systems and on posttranslational modifications other than ubiquitination. In this regard, we recently reported that in human T lymphocytes, phosphorylation of IRF-1 by the IκB kinase ε negatively affected IRF-1 activity (29). Like the classical example of phosphorylation-dependent ubiquitination and proteasomal degradation of the NF-κB inhibitor IκBα, we are currently investigating the relationship between IRF-1 phosphorylation and ubiquitin-mediated degradation.

IRF-1 ubiquitination by HDM2 is specifically increased during HIV-1 infection in the presence of increasing amounts of Tat and is mediated by the formation of a trimeric complex between Tat, IRF-1, and HDM2, as demonstrated by coimmunoprecipitation analysis (Fig. 2). A deletion of IRF-1 lacking the C-terminal 34 aa (IRF-1^291^) was still able to bind HDM2 through the N-terminal region of IRF-1, but under these conditions, IRF-1^291^ was not degraded, consistent with previous observations that the HDM2 docking site is in the N-terminal portion of IRF-1 (32), while the C-terminal 34 aa of IRF-1 is required for Tat interaction (37). The exact mechanism by which HDM2 stimulates IRF-1 ubiquitination and degradation and how Tat exploits HDM2 activity remain to be established. Indeed, whether HDM2 is a true monoubiquitination E3 ligase or a polyubiquitination E3 ligase has not been determined (60). Whether Tat uses an E4 ligase or whether high levels of HDM2 are responsible for IRF-1 polyubiquitination in infected and Tat-expressing cells remains to be assessed. Indirect evidence supports both mechanisms: p300/CBP, the histone acetyltransferase with E4 ligase activity, has been shown to mediate polyubiquitination of p53 (61) and is also used by Tat to target Tip60 for polyubiquitination and degradation (62). p300/CBP also binds to IRF-1 through a domain distinct from the region of IRF1 involved in Tat interaction (63). On the other hand, a substantial increase in HDM2 expression occurs in HIV-1-infected cells, thus supporting a dose-dependent effect of HDM2 (60) in IRF-1 degradation.

Tat itself has been reported to be a substrate of HDM2, although Tat ubiquitination does not result in a change in Tat stability. On the contrary, HDM2 acts as a positive regulator of Tat-mediated transactivation, and Tat K63 ubiquitination is required for efficient replication of HIV-1 (44). While both Tat and IRF-1 are substrates of HDM2 activity, the mechanistic basis and/or kinetics of these regulatory interactions between Tat, HDM2, and IRF-1 remain to be established. We observed a dose-response effect of Tat on IRF-1 degradation, suggesting that to degrade IRF-1, discrete amounts of Tat are required. This conclusion is also supported by findings in infected Jurkat T cells where ubiquitination and modulation of IRF-1 expression correlated with Tat and HDM2 levels (Fig. 7). We speculate that there may be differential usage of HDM2 by Tat, depending on the extent of Tat expression and/or stage of infection. Thus, by recruiting HDM2, Tat may both increase its activity on the HIV-1 LTR and inactivate a transcriptional protein involved in the host antiviral response (Fig. 8).

The interplay between IRF-1, Tat, and HDM2 may provide a selective advantage to HIV-1 replication. Consistently, we have observed a substantial increase in HIV-1 replication, measured by p24 accumulation, when IRF-1 expression is constitutively knocked out in T cells (data not shown). This is not surprising since IRF-1 represents a network hub in the regulation of the host antiviral, immunomodulatory, and growth modulatory functions. More specifically, the antiviral activities of IRF-1 have been reemphasized by interferon-stimulated gene(ISG) expression screening studies that identified IRF-1 as a potent antiviral effector that inhibited a broad range of viruses, including HIV (20, 22). Consistent with these observations, many ISGs are directly activated by IRF-1 after viral infection (18, 64).

IRF-1 also exerts a number of functions beyond its antimicrobial effects. By targeting IRF-1, Tat may regulate cell growth by inhibiting p21^WAF/CIP1^ and CDK2. In this respect, the loss of the G_1_/S checkpoint associated with the loss of *p21*^WAF/CIP1^ gene expression in HIV-infected cells provides a selective advantage for HIV-1 by allowing viral transcription and replication (65). Thus, the targeting of IRF-1 by HIV-1 Tat again illustrates that a single viral protein can modulate a number of cellular pathways, thus contributing to a replicative advantage for the virus.

## MATERIALS AND METHODS

### Plasmids, transient transfection, and reporter gene assay.

CMVBL, CMVBL IRF-1, and mutant CMVBL IRF1^291^ and CMV-Tat expression vectors have been described previously (37, 66). Flag-tagged IRF1 (Flag-IRF1) was obtained by PCR from CMVBL IRF1 and inserted into pCMV2-Flag (CMV stands for cytomegalovirus) (Clontech Laboratories, Inc.) expression vector using HindIII and XbaI restriction enzymes. Flag-tagged Tat (Flag-Tat) was obtained by *de novo* gene synthesis Gene-Script and then cloned in pCMV2-Flag (Clontech) using BamHI and EcoR1; pTat^C22G^ was generated by site-directed mutagenesis (QuikChange; Stratagene, Cedar Creek, TX) using Flag-Tat as the substrate according to the manufacturer’s protocol. Mutated clones were fully sequenced after identification. pEGFP-C2-Hdm2 (EGFP stands for enhanced green fluorescent protein), p21/WAF/CIP1 luciferase reporter gene and pCDNA3.1 Ub-His(6x) were generous gifts of G. D’Orazi and T. Haas. IRF-1-responding luciferase reporter constructs pISRE-TA was from Clontech. The constructs for p3500 (encoding the entire IRF-1 promoter from −3400 bp to +168 bp) cloned upstream of the luciferase reporter gene was a generous gift of Richard Pine.

Transient transfections were performed using JetPei reagent (Polyplus Transfection SA, Illkirch, France) or the calcium phosphate transfection system (Life Technologies, Invitrogen Corporation, Carlsbad, CA) according to the manufacturer’s protocol. The amounts of transfected DNA were normalized by using an empty vector.

Reagents from Promega (Promega Corporation, Madison, WI) were used to assay extracts for dual-luciferase activity in a Lumat LB9501 luminometer (E&G Berthold, Bad Wildbad, Germany).

### Cell culture and reagents.

J-Lat Tat-GFP cells (clone A2/A72) from Eric Verdin (45) was obtained through the NIH AIDS Reagent Program, Division of AIDS, NIAID, NIH. Jurkat, Jurkat clone A2/A72, and HEK293 cells were grown in RPMI 1640 medium and Dulbecco’s modified Eagle’s medium (DMEM) (Bio-Whittaker, Cambrex Bio Science, Verviers, Belgium), containing 10% fetal calf serum (FCS) and antibiotics. Human peripheral blood mononuclear cells (PBMCs) from healthy donors were isolated by Ficoll-Hypaque gradient centrifugation, and the CD4^+^ T cell population was purified by negative selection using magnetic beads (Miltenyi Biotech GmbH, Bergisch Gladbach, Germany), as previously described (29). Recovered cells were >96% CD4^+^, as determined by fluorescence-activated cell sorter (FACS) analysis. The cells were cultured in RPMI 1640 medium (Bio-Whittaker, Cambrex Bio Science) containing 20% FCS and antibiotics and activated with anti-CD3 monoclonal antibodies (MAbs) (R&D Systems, Minneapolis, MN). MG132 (Sigma) was used at 50 µM, cycloheximide (CHX) (Sigma) was used at 25 µg/ml, and tumor necrosis factor alpha (TNF-α) was used at 10 ng/ml.

### HIV stock preparation and infection.

Replication-competent dual-tropic virus was generated by calcium phosphate-mediated transient transfection of HEK293 cells with the pHXB2R molecular clone. Virus-containing supernatant was filtered, frozen in aliquots at −70°C, and titrated on TZM-bl cells. Jurkat and primary CD4^+^ T cells were inoculated with HIV-1/HXB2 at a multiplicity of infection of 0.05 50% tissue culture infective dose (TCID_50_) per cell, as previously described (37).

### Quantitative real-time reverse transcription-PCR.

Total RNA extracted using the RNeasy total RNA extraction kit (Qiagen) was treated with RNase-free DNase (Qiagen) and then reverse transcribed with High Capacity cDNA reverse transcription kit (Applied Biosystems) according to the manufacturer’s instructions. cDNA was subjected to quantitative real-time PCR on ABI 7000 sequence detection system (PE Applied Biosystems, Warrington, United Kingdom) by using SYBR green PCR master mix (Applied Biosystems). Primers used for quantitative real-time reverse transcription-PCR (qRT-PCR) were IRF-1 forward primer 5′-AGCTCAGCTGTGCGAGTGTA-3′ and reverse primer 5′-CATGACTTCCTCTTGGCCTT-3′ and Tat/Rev forward primer 5′-CTTAGGCATCTCCTATGGCAGGAA-3′ and reverse primer 5′-GGATCTGTCTCTGTCTCTCTCTCCACC-3′. Transcript levels were normalized to glyceraldehyde-3-phosphate dehydrogenase (GAPDH) (forward, 5′-GGGTGTGAACCATGAGAAG-3′; reverse, 5′-GCTAAGCAGTTGGTGGTGC-3′) as an internal control and expressed as fold increase according to the Δ*C_T_* methods (means ± standard deviations).

### Coimmunoprecipitation, Western blot analysis, and protein quantifications.

Total protein extracts (whole-cell extracts [WCE]) were prepared and subjected to Western blot analysis or immunoprecipitation, as previously described (39). Briefly, for coimmunoprecipitation, 300 µg of WCE was incubated with 1 µg of polyclonal anti-IRF-1 antibody (sc-13041; Santa Cruz Biotechnology Inc., Santa Cruz, CA) overnight at 4°C, and then Ultralink immobilized protein A/G-Sepharose (Pierce Biotechnology, Rockford, IL) was added for 2 h at room temperature. Alternatively, anti-FlagM2 antibody cross-linked resin (Sigma) was added to lysate and processed according to the manufacturer’s instructions. After extensive washing, immunoprecipitates were eluted by boiling the beads for 5 min in 2× sodium dodecyl sulfate (SDS) sample buffer and then subjected to Western blot analysis. IRF-1, IRF-1 deleted form (IRF-1^291^), HDM2, GFP-tagged, and Flag-tagged proteins were detected by anti-IRF1 (sc-497; Santa Cruz Biotechnology), anti-IRF1 (sc-13041; Santa Cruz Biotechnology), anti-HDM2 (oncogene Ab2 clone 2A10), anti-Flag M2 (Sigma), and anti-GFP (Santa Cruz Biotechnology) primary antibodies, respectively. Polyclonal antibody against Tat was a generous gift of B. Ensoli. Anti-UbK48 (Apu2; Millipore), anti-UbK63 (HWA4C4; eBiosciences), anti-p21 (Santa Cruz Biotechnology), anti-CDK2 (clone AN4.3; Millipore), and anti-actin antibody (Santa Cruz Biotechnology). Secondary antibodies were from Calbiochem (San Diego, CA). The levels of IRF-1 protein relative to the levels of endogenous actin protein were quantified on Western blots using a Fluor-S Multi-Imager (BioRad) system and Quantity One Fluor S software.

### Nickel capture assay.

HEK293 cells were plated in 10-cm dishes and cotransfected with expression plasmids encoding ubiquitin-His(6×), Flag-Tat, and full-length or mutant IRF-1 (IRF-1 or IRF-1^291^). The cells were harvested 24 h after transfection, and 20% were lysed and used for direct Western blot analysis as previously described (39). The remaining cells were lysed in 6 ml of highly denaturing buffer A (6 M guanidium-HCl, 10 mM Tris-HCl [pH 8], 100 mM Na_2_HPO_4_/NaH_2_PO_4_ [pH 8.0], 5 mM imidazole, and 10 mM β-mercaptoethanol). The lysates were sonicated to reduce the viscosity. His-Ub-conjugated proteins were purified by nickel chromatography upon incubation with 70 µl of nickel-NTA-agarose beads (Qiagen) overnight at 4°C. The beads were then washed once in buffer B (8 M urea, 100 mM Na_2_HPO_4_/NaH_2_PO_4_ [pH 8.0], 10 mM Tris-HCl [pH 8], and 10 mM β-mercaptoethanol), twice in buffer C (8 M urea, 100 mM Na_2_HPO_4_/NaH_2_PO_4_ [pH 6.3], 10 mM Tris-HCl [pH 6.3], 10 mM β-mercaptoethanol, and 0.2% Triton X-100), once in buffer C plus Triton 0.1%. His-Ub-conjugated proteins were then eluted with 50 µl of buffer D (0.15 M Tris-HCl [pH 6.7], 30% glycerol, 0.72 M β-mercaptoethanol, 5% SDS supplemented with 200 mM imidazole) while being stirred for 20 min at room temperature. Sample buffer was added, and the supernatants were subjected to SDS-PAGE and Western blot analysis.

### Statistical analysis.

Significant differences between experimental points measured by qRT-PCR and luciferase assays were assessed by using the Student-Newman-Keuls posttest following significant (*P* < 0.05, *P* < 0.01, and *P* < 0.001) repeated-measure analysis of variance (ANOVA).
